# Bioceramic Resonance Effect on Meridian Channels: A Pilot Study

**DOI:** 10.1155/2015/769546

**Published:** 2015-07-06

**Authors:** Ting-Kai Leung, Wing P. Chan, Chen-Jei Tai, Ting-Pin Cho, Jen-Chang Yang, Po-Tsung Lee

**Affiliations:** ^1^Department of Radiology, Taipei Hospital, Ministry of Health and Welfare, No. 127 Su Yuan Road, Hsinchuang, New Taipei City 242-13, Taiwan; ^2^Department of Radiology, Wan Fang Hospital, Taipei Medical University, No. 111, Sec. 3, Xinglong Road, Taipei 116, Taiwan; ^3^Graduate Institute of Biomedical Materials and Tissue Engineering, College of Oral Medicine, Taipei Medical University, No. 250, Wu-Hsing Street, Taipei 110, Taiwan; ^4^Department of Radiology, Shanghai East Hospital, 1800 Yuntai Road, Pudong New Area, Shanghai 200123, China; ^5^Department of Traditional Chinese Medicine, Taipei Medical University Hospital, No. 252, Wu Hsing Street, Taipei 110, Taiwan; ^6^Metal Industries Research & Development Centre, 1001 Kaonan Highway, Kaohsiung 811, Taiwan; ^7^School of Dentistry, College of Oral Medicine, Taipei Medical University, Taipei 110, Taiwan; ^8^Department of Photonics and Institute of Electro-Optical Engineering, National Chiao Tung University, 1001 Ta Hsueh Road, Hsinchu 300, Taiwan

## Abstract

Bioceramic is a kind of material which emits nonionizing radiation and luminescence, induced by visible light. Bioceramic also facilitates the breakup of large clusters of water molecules by weakening hydrogen bonds. Hydrogen bond weakening, which allows water molecules to act in diverse ways under different conditions, is one of the key mechanisms underlying the effects of Bioceramic on biophysical and physical-chemical processes. Herein, we used sound to amplify the effect of Bioceramic and further developed an experimental device for use in humans. Thirteen patients who suffered from various chronic and acute illnesses that severely affected their sleep patterns and life quality were enrolled in a trial of Bioceramic resonance (i.e., rhythmic 100-dB sound waves with frequency set at 10 Hz) applied to the skin surface of the anterior chest. According to preliminary data, a “Propagated Sensation along Meridians” (PSM) was experienced in all Bioceramic resonance-treated patients but not in any of the nine control patients. The device was believed to enhance microcirculation through a series of biomolecular and physiological processes and to subject the specific meridian channels of Traditional Chinese Medicine (TCM) to coherent vibration. This noninvasive technique may offer an alternative to needle acupuncture and other traditional medical practices with clinical benefits.

## 1. Introduction

**Figure 1:**
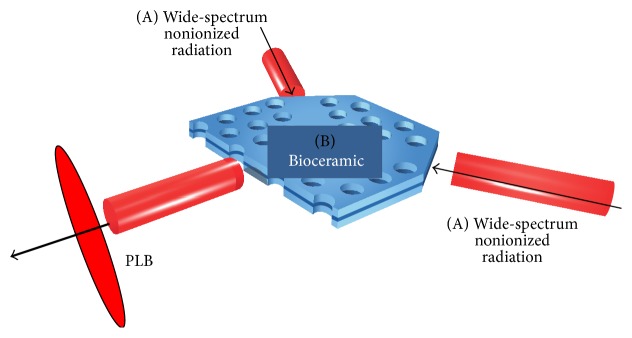
Conceptual picture of photoluminescence (PL) emission from the Bioceramic material (B) in response to excitation by visible light (A).

Bioceramic is material, modified from high-performance far infrared ray (FIR) emitting material, and possesses the characteristics of emitting nonionized radiation spectrum and visible light-induced luminescence [1–4]. In preclinical and clinical studies, we showed that Bioceramic-emitted light improves microcirculation, glucose control, and skin conductance at the acupuncture points of Traditional Chinese Medicine [1–4]. Photoluminescence is a special type of luminescence. Photoluminescence is light emitted from material in response to light energy absorption and the interaction between electromagnetic radiation and matter [5–7]. Photoluminescence of Bioceramic (PLB) is the combination of Bioceramic and visible light ray [6, 7], when a visible light source (e.g., light-emitting diodes (LEDs)) without necessary additional thermal effect can propagate the effect of Bioceramic on living animal and human being (Figure 1). As far as we know, our publications are the first to report the phenomenon of photoluminescence of Bioceramic (PLB) material emission, which can produce its effect at long distance via visible light rays [1–4]. PLB emission can enhance microcirculation and “Propagated Sensation along Meridians” (PSM) of acupuncture as described previously but in less than 20% of cases [4]. To examine the possible health promotion effects of Bioceramic technology, we reviewed the publications of Professor Wei-Kung Wang and his colleagues. Wang et al. stated that each pulse wave in the arterial system can be broken down into many harmonic frequencies based on an individual's heart beat frequencies. Based on Wang's deduction, the source of the twelve meridian channels is the individual's heart beat and harmonic frequencies. Acupuncture points and meridian channels are thought to be closely related to the microcirculation [7–10] (explained below). Besides visible light, we also using sound frequency to construct Bioceramic resonance device. According to our new observations and the so far unpublished data, the sound waves have long-distance effects because they can move long distances and also bend around corners.

To choose the suitable frequency for our experiment, we reviewed previous articles and studies on the effects of different frequencies. A study by Rasmussen found that frequencies from 1 to 20 Hz are the most effective [11]. We selected 10 Hz (mean value of 1 to 20 Hz) as the first frequency setting to test our Bioceramic resonance device for this study.

In this study, we describe the newest design of our Bioceramic device and the effect of resonance on the meridian channels of the human body and discuss the possible applications.

## 2. Materials and Methods

The Bioceramic powder used in this study (obtained from Bioenergy Laboratories, Bioenergy Development Ltd., Taoyuan, Taiwan) was composed of microsized particles containing several ingredients, mainly elemental components [1–4]. The average emissivity of the ceramic powder was 0.98 at wavelengths of 6~14 *μ*m (determined using a CI SR5000 spectroradiometer), indicating an extremely high far infrared ray emission rate (Figure 1). This ceramic powder can induce many physical, chemical, and biological effects at room temperature without the need for direct contact [1–4].

### 2.1. Photoluminescent Bioceramic (PLB) Material and Bioceramic Irradiation Combined with Sound Waves of Frequency (10 Hz)

**Figure 2:**
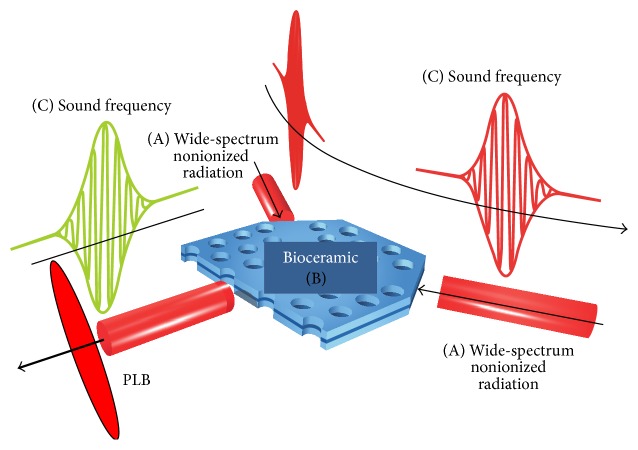
The combination of PL emission and acoustic radiation (sound waves) (C).

Photoluminescent substances are materials that absorb light energy and then release that energy in the form of light. Photoluminescence obeys the laws governing the interaction between electromagnetic (EM) radiation and matter. Bioceramic material absorbs a portion of the EM spectrum (including near, middle, and far infrared wavelengths) and emits lower energy wavelengths, providing for our purpose a useable visible light source. In the present study, these light sources were visible light-emitting diodes (LEDs), emitting light energy in the range of wavelengths between 480 nm and 780 nm. The level of illumination was strictly controlled at 500 lux, thus avoiding thermal effects on the skin of the participants. In addition, a rhythmic 100-dB sound with frequency 10 Hz was projected through a Bioceramic membrane (consisting of 10% Bioceramic material mixed with silicone rubber) onto the skin surface of the anterior chest wall to achieve resonance with the tissues of the whole human body. The concept of emission from a Photoluminescent Bioceramic (PLB) material acting in concert with rhythmic sound waves is shown in Figure 2.

#### 2.1.1. Experimental Methods to Test the Effect of the Bioceramic Device on Hydrogen Bonding

Procedures used to determine the key effect and mechanism of the Bioceramic device are as follows.

*(a) Fourier Transform Infrared Spectroscopy (FT-IR). *FT-IR is one of the most precise methods used to study H-bonds. Control double distilled water (DDW) and Bioceramic-irradiated DDW treated for 1 h were prepared for infrared measurement. Aliquots were sealed between a pair of CaF_2_ windows* (Beckman FH-01)* with a thin Teflon spacer approximately 150 *μ*m in thickness. The infrared spectrum was recorded at 25°C at a resolution of 2 cm^−1^ using a Nicolet Magna 550 spectrometer equipped with a* dTGS detector*. An average of 32 scans were carried out and merged for each sample. (Detection of an effect on hydrogen bonding requires a large number of scans and high resolution of 2 cm^−1^.) The data were then calculated from the absorption spectrum in the far infrared ray region (3400 cm^−1^).

*(b) Coating of the Bioceramic by Sputter Deposition*. Bioceramic with purity of 100% was sputtered onto the target quartz surface. Sputtering was performed using a closed field unbalanced magnetron sputtering system supported by four vertical cathodes at intervals of 90° (developed by Metal Industries Research & Development Centre, Kaohsiung, Taiwan). The target dimensions (quartz) were 300 mm × 100 mm × 10 mm. The sputtering current was 1 Amp. (370 Watt., 370 Volt.). The power was supplied by an* ENI RPG-100 *pulse generator with DC pulse, frequency of 50 kHz, and pulse width of 1056 ns. The Argon (Ar) flow rate was maintained at 30 standard cubic centimeters per minute (sccm) with working pressure at 3 × 10^−3^ Torr. The coating architecture was single layer and no buffer layer. The surface coating thickness was 22 nm for optimal outlook translucency. The light pump used the photoluminescence spectra (PL Spectrum), with detection band of <1 *μ*m. After absorbing visible light, the Bioceramic material emitted a slightly lower energy light signal. The three different photon energy sources included (1) a 488-nm visible light laser (blue), (2) 532-nm visible light laser (green), and (3) 633-nm visible light laser (red). The light was projected onto the translucent quartz surface coated with Bioceramic material.

#### 2.1.2. Construction of the Bioceramic Resonance Device and Trial of the Device in Humans

*Candidates*. Thirteen candidates (six females and seven males) consented to participate in this clinical trial. All were adults with different health issues. The study protocol was approved by the Human Subjects Committee at the Taipei Hospital (Ministry of Health and Welfare), New Taipei City, Taiwan (approval number: TH-IR-0014-0001). The patients all suffered from insomnia in conjunction with other symptoms, such as dyspepsia, migraine, anxiety, low back pain with radiculopathy, poststroke paralysis of the extremities, and leg swelling due to deep vein thrombosis. As aforementioned, since sound can enhance the effect of the Bioceramic device (Figure 2), we developed instrument of Bioceramic Resonance (BR) for human trials, capable of producing a sound frequency resonance effect. The vibration source was at about 50 cm from the skin surface of anterior chest wall and the average sound level was 100 dB. The candidates received Bioceramic resonance with rhythmic sound frequency set at 10 Hz. Clinical observation and subjective descriptions (such as time and duration of PSM experiences over the 1-hour experimental period) were recorded at posttreatment interviews to assess possible changes in sensation throughout the period.

On the other hand, nine candidates with insomnia received the usual light and 10-Hz sound frequency but without Bioceramic resonance.

## 3. Results 

### 3.1. Bioceramic Irradiation Affects Hydrogen Bonding

**Figure 3:**
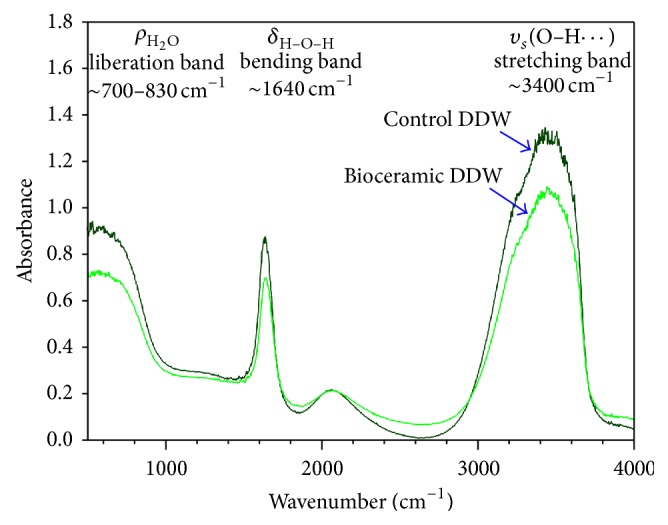
Area under the absorbance curve of Bioceramic-irradiated DDW in the FIR region (3400 cm^−1^) was significantly decreased, compared to that of the control nonirradiated DDW.

**Figure 4:**
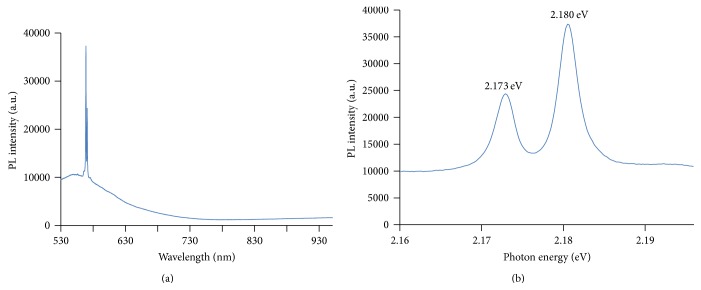
The c-FIR material coated quartz surface absorbed visible laser light (488 nm) and emitted slightly lower energy light signals. The decrease from input to output photon energy (in eV) was 2.541 to 2.173 and 2.180, respectively.

**Figure 5:**
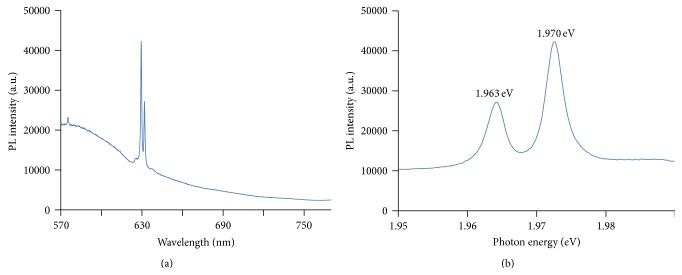
After absorbing visible laser light (532 nm), the coated surface emitted slightly lower energy light signals. The decrease from input to output photon energy (in eV) was 2.331 to 1.963 and 1.970, respectively.

**Figure 6:**
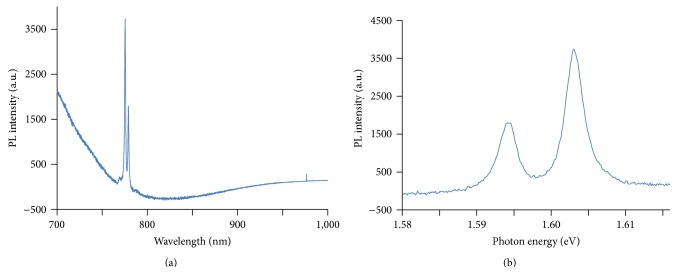
After absorbing visible laser light (633 nm), the coated surface emitted slightly lower energy light signals. The decrease from input to output photon energy (in eV) was 1.960 to 1.592 and 1.599, respectively.

Absorbance of light in the far infrared ray (FIR) region (3400 cm^−1^) by Bioceramic-irradiated DDW was significantly below that of control DDW. This result indicates a hydrogen bond weakening effect of Bioceramic irradiation on DDW (Figure 3). The projection of photons with wavelengths 488 (blue; Figures 4(a) and 4(b)), 532-nm (green) (Figures 5(a) and 5(b)), and 633 nm (red; Figures 6(a) and 6(b)) across the translucent quartz coated with Bioceramic material resulted in a definite pre- to postexcitation difference in the energy of the emitted photoluminescence signal.

### 3.2. Energies of the Emitted Photons

**Figure 7:**
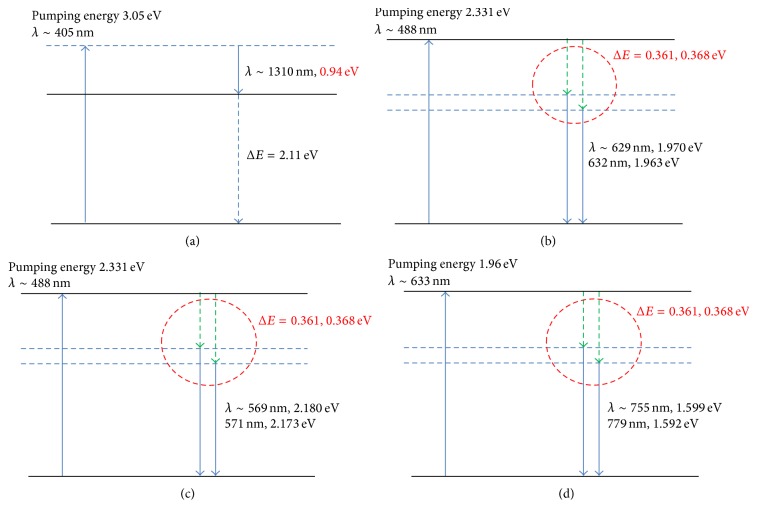
Energy difference: Δ*E* = 0.361 eV (3435 nm) and 0.368 eV (3369 nm) are shown, and dotted lines represent assumed transitions.

The energy differences between the peaks of photon emission after Bioceramic material absorption of the three different wavelengths of the visible light were 0.361 eV and 0.368 eV below that of the original excitatory light sources (Table 1; Figure 7).

### 3.3. Clinical Observations Relevant to Bioceramic Resonance

**Figure 8:**
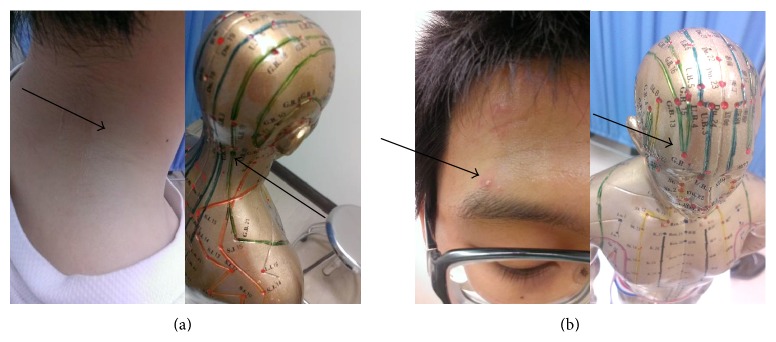
Subjective description of PSM by a candidate (arrows on left sides) who virtually traced the GB, one of the twelve main meridian channels of TCM (arrows on right sides).

The thirteen individuals exposed to Bioceramic irradiation with 10-Hz rhythmic sound vibrations (but not the nine controls exposed to the usual light and 10-Hz rhythmic sounds, without Bioceramic irradiation) reported experiencing a “Propagated Sensation along Meridians” (PSM). PSM as described in our previous study was reported in response to using PLB on acupuncture points, but the proportion of participants experiencing PSM was lower than 20% [9]. Bioceramic treatment elicited PSM on the skin's surface in lines that correspond to meridian lines on the skin's surface or the twelve main meridian channels according to Traditional Chinese Medicine (TCM) (Table 2; Figures 8(a) and 8(b)) [1, 9].

## 4. Discussion

### 4.1. Reasons for the Effect of Bioceramic Irradiation on Water Properties

We investigated the weakening of the hydrogen bonds of water after Bioceramic irradiation and the resulting effects on physical, biological, and medical properties of water. Fourier transform infrared spectroscopy (FT-IR) was used herein to explore the Bioceramic irradiation-induced change in hydrogen bonding; in addition, capillary viscometers, gas chromatographs (GC), differential scanning calorimetry (DSC), contact angle analysis, Franz cells, high-performance liquid chromatography (HPLC), and capillary electrophoresis analysis have been used to evaluate other physical characteristics, such as viscosity, volatility, temperature of water crystallization, surface tension, diffusion, solubility of solid particles, and changes in the pH of acetic acid [1–4]. The Bioceramic irradiation caused the decrease in viscosity and surface tension (contact angles) of water, but the increase in the solubility of solid particles in water, temperature of water crystallization, and acidity of acetic acid. The weakening of water hydrogen bonds corresponds to the increase in microcirculation described in our previous reports. At the three excitation wavelengths, the energy differences between the pumping photons and the emitted photons were 0.361 eV (corresponding to wavenumber at 2910 cm^−1^) (Figure 3 and Table 1) and 0.368 eV (corresponding to wavenumber at 2960 cm^−1^; Figure 3 and Table 1), which lie within the absorption spectrum of FT-IR. The energy of this characteristic irradiation might lead to the break-up of large water clusters in DDW (hydrogen bond strength 5–30 kJ/mole), reduction in the volume charge density, and formation of effective dipoles that could lead to a decrease in absorbance signals [12–14].

Water possesses important properties required for life-giving processes [15, 16]. Three or four strong hydrogen bonds are needed to form clusters of water molecules, and it is this feature of hydrogen bonding that is responsible for the special properties of water. Weakening of hydrogen bonds allows water to act in diverse ways under different conditions [17]. Hydrogen bond weakening is the basis for the biophysical and chemical effects of Bioceramic irradiation reported in our previous publications [1–4].

### 4.2. Lessons from Human Trial Studies That Can Be Applied to Therapy

Since all participants who received Bioceramic Resonance experienced “Propagated Sensation along Meridians” (PSM), our results seem to suggest that Bioceramic irradiation stimulates the twelve main meridian channels according to Traditional Chinese Medicine (TCM) (Table 2 and Figures 8(a) and 8(b)) [1, 4]. PSM becomes apparent from one to about twenty minutes after Bioceramic Resonance is initiated and as it moves along the meridiansof an acupuncture point. PSM is propagated along the meridian channels during acupuncture by two assumed mechanisms: (1) neuromodulation [18–20] and (2) histamine release due to degranulation of mast cells, which leads to capillary dilation, increase in blood perfusion, and interstitial fluid [20]. Although the existence of meridian channels as described in the ancient Chinese medical textbook (The Yellow Emperor's Inner Classic) published over 2500 years ago is scientifically unproven, our Bioceramic resonance device will allow the gathering of more concrete and objective evidence of PSM. To explain the PSM response to Bioceramic irradiation, we reviewed the past literature [8, 9] on the origin of meridian channels. Previous studies used Fourier transform analysis to decompose the frequency range of signals (such as pulsatile pressure due to heartbeat and rate of radial or femoral artery blood flow that change over time) into eleven harmonic sound frequencies.

The equation is as shown below:

(1) $$\begin{array}{c} P\left(t\right)=\overset{11}{\underset{n=1}{\sum }}\left(b_{n}\sin\left(nNt\right)\right) \end{array}$$

(*P*: pressure; *t*: time; *n*: number of harmonic sound frequencies; *b*_*n*_: pressure of the number “*n*”; *N*: original heartbeat frequency; *P*(*t*): pressure change in time; ∑_*n*=1_^11^: adding from 1st to 11th harmonic sound frequency; *nN*: frequency of the number “*n*”).

Based on Wang's deduction, the energy of a twelfth frequency is the sum of the energies of the eleven harmonic frequencies. According to series of publications by Wang et al., the harmonic rhythmic sound frequencies of the heartbeat are the main frequency components of the propagated pressure wave and correspond to the twelve meridian channels of TCM. Acupuncture points and meridian channels are thought to be closely related to microcirculation [7–10]. On the other hand, rhythmic low frequency sound waves are characterized by longer wavelengths, weaker attenuation, and greater depth of penetration than high frequency sound waves. It was reported that rhythmic low frequency sound travels more efficiently through human tissues. While acupuncture is being administered, a rhythmic sound of 2–15 Hz could produce vibrations that resonate with the vibrations of human tissues or organs [21]. In other words, meridian channels have different harmonic frequencies all based on the individual's heartbeat frequency. Another hypothesis by Zhang et al. suggests that meridian channels course through subcutaneous tissues with fluid flowing within them [1, 22], and Bioceramic irradiation weakens hydrogen bond strength in the fluid thereby enhancing microcirculation. To answer the question of why sensation is propagated along meridians, we propose that rhythmic sound waves (10 Hz) and their different harmonic frequencies produce vibrations that resonate with the vibrations of the meridian channels of TCM and that the combined effects of Bioceramic irradiation and photoluminescence on hydrogen bond weakening enhance microcirculation, which resonate with harmonic frequencies of the individual's heartbeat. We suggest that tissues of interstitial fluid channels that fail to resonate with heartbeat harmonic frequencies will be enforced by Bioceramic effect and may enhance mast cell degranulation and histamine release along the specific meridian channels. It is further causing capillary dilation, increased blood and interstitial fluid circulation, and simultaneous continuous nerve signals to regulate the nervous system or neuromodulation. We propose that this train of nerve impulses is the basis for PSM that is provoked by Bioceramic resonance. Even though the above hypothesis needs further elaboration, rhythmic sound frequency resonance begins a new era in TCM and its therapeutic concepts. The Bioceramic resonance technique faces two major challenges to its acceptance by mainstream medical science. First, the pathways of meridian channels must be demonstrated anatomically and second the correspondence of different meridian channels to specific vibration frequencies must be demonstrated anatomically [22]. We hope that more support, constructive criticism, and comments will be forthcoming to facilitate more successful future studies.

## 5. Conclusion

The Bioceramic resonance device combines nonionizing radiation with rhythmic sound frequencies (10 Hz) to provoke hydrogen bond weakening and facilitate microcirculation improvement. This combination resulted in effective coherent vibrations within human tissue that elicited PSM along TCM meridian lines in our thirteen candidates. The noninvasive Bioceramic resonance technique is potentially an alternative method to other Traditional Chinese methods (such as needle acupuncture or moxibustion). More experiments should be performed in the expectation that this method will improve treatment of many illnesses.

## Figures and Tables

**Table 1:** Calculation of energy differences between the absorbed and emitted infrared photons.

	PL energy	First excited photon energy (*E*1)	Second excited photon energy (*E*2)	Δ*E*1 (eV) = *E* − *E*1	Δ*E*2 (eV) = *E* − *E*2
488 nm laser	2.541 eV	2.180 eV	2.173 eV	0.361 eV	0.368 eV
532 nm laser	2.331 eV	1.970 eV	1.963 eV	0.361 eV	0.368 eV
633 nm laser	1.960 eV	1.599 eV	1.592 eV	0.361 eV	0.368 eV

**Table 2:** Candidate characteristics and clinical observations after Bioceramic resonance treatment.

Chief complaints	Sex	Age	Location(s) of PSM	Duration of PSM after Bioceramic resonance treatment	Observations during the one-hour period following treatment
Dyspepsia	M	25	Yangming Stomach Channel at bilateral sides of the throat, chest, abdomen, and lower extremities	Within one minute	No further change

Poor appetite	F	32	Yangming Stomach Channel at bilateral sides of the throat, chest, and abdomen	About five minutes	No further change

Posttraumatic head injury (right side), complicated by intermittent migraine	F	55	Shaoyang Gallbladder Channel at right side of head, posterior neck, and upper lateral chest	About two minutes	No further change

Insomnia	M	61	Jueyin Pericardium Channel at bilateral ventral sides of hands and arms	About ten minutes	No further change

Insomnia	F	36	Taiyang Bladder Channel and Shaoyang Gallbladder Channel on bilateral sides of the head and posterior neck	About five minutes	No further change

Migraine	M	62	Shaoyang Sanjiao Channel of the left upper arm	About six minutes	No further change

Anxiety and insomnia	M	36	Shaoyang Sanjiao Channel at the bilateral sides of lateral scalp of head	About 10 minutes	No further change

Benign facial tremor, left	F	52	Shaoyang Gallbladder Channel of the bilateral upper arms	About 10 minutes	No further change

Low back pain with bilateral posterior leg radiculopathy	M	45	Taiyang Bladder Channel of the buttock and bilateral thighs and legs	About 5 minutes	No further change

Old hemorrhagic stroke with facial weakness and paralysis of the right upper and lower arm	M	60	Shaoyang Gallbladder Channel of the right posterior neck	About 20 minutes	No further change

Insomnia	F	40	Taiyang Bladder Channel of the occipital head	About 10 minutes	No further change

Insomnia	F	45	Shaoyang Sanjiao Channel of the right hand to arm	About 5 minutes	No further change

Deep vein thrombosis of the bilateral lower legs Posttraumatic injury of the right great toe with poor wound healing	M	49	Jueyin Liver Channel at the dorsomedial side of the right foot	About 15 minutes	No further change
